# Supplementary material for: Learning curve and surgical outcome of robotic assisted colorectal surgery with ERAS program
Source: Sci Rep. 2022 Nov 29;12:20566. doi: 10.1038/s41598-022-24665-w (PMC9709162; doi:10.1038/s41598-022-24665-w)
Supplement: Supplementary file 2 — Supplementary Information 2. [file 41598_2022_24665_MOESM2_ESM.zip › RAL Raw data-Ñ[▒K/077/HS_ñw╝╨░O▒Kñσ.pdf]

正式摘要(電子簽章：000000215426566)	【讀取】憑證	
W52 / W52 ☐ 英文標題(注意:無英文姓名-請補病人基本檔!)		

臺中榮民總醫院
出院病歷摘要

轉入號 : 轉出號 :

[redacted]

**(8)**流水編號	
**(9)**入院日期	110/04/21 (2021) 直腸外科 W52 - 161
**(10)**轉科日期	
**(11)**出院日期	110/04/26 (2021) 住院天數計 5 日

診斷

- 入院 : 1. Rectal tumor
- 出院 : 1. Rectal tumor
  - s/p robotic low anterior resection on 2021/04/22

主訴

- bloody stool for one month

病史

- This 60 year-old female had history of
  1.Hypertension under valsartan
  2.Dyslipidemia under atovastatin
  3.Appendicitis s/p laparoscopic appendectomy
  She had blood in stool since March.2021, and went to Tung H., Coloscope showed malignant there. She went to our CRS OPD for help. Abd CT showed 1.2cm rectal tumor within colonic or rectal wall. Under impression of rectal cancer, T1/T2N0M0, she was admitted to our ward for surgical intervention and further treatment.

體檢發現

-
  - General appearance :
    - easy looking , conscious clear , without cardiopulmonary distress
    - Vital signs自動帶入date 體溫(BT) 脈搏(P) 呼吸(R) 血壓(BP) 身高(BH) 體重(BW) BMI
      20210421 36.3℃ 58次/分 18次/分 107/72mmHg 153.4cm 50.4kg 21.4
    - Vital signs: B.P. - mmHg , P.R. 5 /min , R.R. 5 /min
  - HEENT :
    - Eyes: no anemic conjunctiva, no icteric sclera, normal EOM
    - ENT: no otorrhea, no rhinorrhea, no erythematous mucosa, no injected tonsil, no deformity
  - Neck: supple, freely movable, trachea midline,no deformity, no engorged jugular vein , no palpable thyroid or lymph node enlargement
  - Chest:
    - Inspection: Grossly normal, symmetric and fully expansion, no accessory muscle used, no operation scar, no spider angiomata, no gynecomastia
    - Palpation: no palpable mass or nodule, no subcutaneous emphysema, no tenderness
    - Ausculation: clear breathing sound
  - Heart:
    - Percussion: normal heart size
    - Ausculation: regular heart beat, normal S1, S2, no gallop, no murmur
  - Abdomen:
    - Inspection: in
      - flat contour, symetrically, no scar, no stria, no dilated veins, no rashes
    - Ausculation: normoactive bowel sound, no abdominal bruits
    - palpation: soft, no muscle guarding or rigidity, no local tenderness, no rebounding pain, no palpable mass, no palpable pulsation

- - Percussion: no shifting dulllness, no hepatomegaly, no splenomagaly
-

Peripheral pulsation :	CA	BA	RA	FA	PA	PTA	DPA
Right side :	++	++	++	++	++	++	++/+++
Left side :	++	++	++	++	++	++	++/+++

- Genito-anus: no tenderness or palpable mass lesion or rectal shelf, normal external genitalia, normal anal tone, brown stool

CRS CONDITION

-
  - Lung

手術日期及方法

- 手術日期及方法20210422 1. Robotic (XI) assisted low anterior resection
  2. Intraoperative colonoscopy : (-).
  3. High ligation (+) : At IMA root (Via robotic clips). IMV was ligated via endoclip.
  4. Take down from the splenic flexure : (-)
  5. Robotic EndoGIA (45-4.3) X2 + EEA (29mm) + OpenGIA (80-3.8) X1 were applied to complete the side-to-end anastomosis
  6. Proximal cut end: 8 cm, distal cut end: 3 cm
  7. No drainage tube was inserted
  8. ADHESION BARRIER FILM was placed below the umbilicus wound
- 手術結果及發現 :
  - 1. A 2 x 2 cm tumor at lower rectum. (DRE : Could not palpate the tumor) 2. Intra-operative scopy : The tumor was identifed at middle-low rectal area. A small polyp was also noted at low rectum. 3. Some adhesion bands were noted below the umbilicus and the pelvic area. It was probably related to the laparoscopic appendectomy surgery. 4. There was no obvious visible liver metastasis, no visible peritoneal seeding 5. No obvious ascites was noted

住院治療經過

- After admission, there was no contraindication for operation and robotic assisted LAR was performed smoothly on 04/22. She tolerated the procedure and no post-op complication was noted. She recovered well and oral intake was smooth since 04/23. Under the stable condition, she was discharged and would be followed-up at OPD.

合併症

- nil

一般檢查

- 累積報告 :

DATE	TIME	ALB	TP	BIL.T	BIL.D	ALKP	AST	ALT	LDH	CHOL	TG	HDL-C	LDL	UA	NH3	AMY
1100422	0900	4.0	6.5			64	21	21		135	57	58	64	3.8		
1100422	2103	3.6		0.7			18	18								

DATE	TIME	WBC	RBC	HGB	HCT	MCV	PLT	BLST	NEUT	BAND	SEG	LYM	MON	EOS	BAS
1100422	0903	4210	3.57	11.5	35.0	98.0	218		50.5			40.9	6.7	0.5	1.4
1100422	2102	9040		10.6	32.7		179		90.4			6.5	2.5	0.3	0.3

DATE	TIME	NA	K	CL	CA	BUN	CREAT	Mg	P	CRP	ESR	PCT	LIPASE	LACTATE
1100422	0900					13	0.85			0.013				
1100422	2103	140	3.7		8.0	10	0.79							

DATE	TIME	GLUCOSE	PAB	TRF	ZN	CU	KETONE	CK	CKMB	PT-P	PT-C	APTT-P	APTT-C	TRO-T
1100422	0910	69												

- 放射線報告 :**檢驗項目 :CT ABDOMEN-SURVEY 報告日期 :1100420**
  主　訴 :
  Result:Y
  Finding:
  Imaging Report Form for Colorectal Carcinoma (AJCC 8th)
  1. Imaging Date: 20210416
  Imaging Modality, Imaging by
  CT scan
  Imaging Protocol: CT scan of abdomen with IV and oral contrast enhancement
  2. Tumor location / Size
  Location:
  Rectum
  Size:
  Measurable: 1.2 cm (largest diameter)
  3. Tumor invasion

- Tumor within colonic or rectal wall
  4. Regional nodal metastasis
  No
  5. Distant metastasis (In this study)
  No or Equivocal
  6. Other findings: > A case of rectal cancer s/p biopsy, for survey
  > Suspicious wall thickening over rectum(axi:60), may be compatible with rectal cancer
  > Bilateral small renal cysts
  > Right small renal stone over lower calyx
  > The liver, gallbladder, pancreas, spleen and visible lung fields are without specific abnormality
  > No significant lymphadenopathy in the abdomen or retroperitoneum is identified
  > DJD change of thoracic and lumbar spine with osteophytes formation.
  Impression:
  Rectal cancer, T1/T2N0M0

特殊檢查

病理報告

- Pending

其它

出院時情況

- 3.改門診治療

出院指示

藥名	劑量	劑量單位	用法	頻次	數量	數量單位	現況	天數	單位
(□)Takepron OD tab 30mg	1	TAB	PO	QDAC	7	TAB	TH	7	天
Ultracet tab	1	TAB	PO	Q6H	28	TAB	TH	7	天

出院計劃

主治醫師 2062F 蔣鋒帆 / 住院醫師 2334J 陳勇先

正式摘要(電子簽章：000000215426566)

W52 / W52

doctorRank:B
